# Cap-assisted 5-cm diameter cold snare treatment for phytobezoars: A retrospective study

**DOI:** 10.1371/journal.pone.0323226

**Published:** 2025-05-07

**Authors:** Jie Liu, Zhang Tao, Wenfeng Pu, Yan Zhang, Zonghan Du, Long Chen, Dan Hu, Yanan Chen, Guobin Li, Lisha Zhang, Yiwen Yu, Fuxia Wei

**Affiliations:** Department of Gastroenterology, The Affiliated Nanchong Central Hospital of North Sichuan Medical College, Nanchong, Sichuan, China; Dalin Tzu Chi Hospital, Buddhist Tzu Chi Medical Foundation, TAIWAN

## Abstract

**Background and aims:**

The current treatment options for phytobezoars include endoscopic therapy, chemical lysis, and surgical treatment. These methods are often less efficient or are associated with more adverse events in large-diameter phytobezoars. This study aimed to evaluate the feasibility, effectiveness, and safety of the cap-assisted 5-cm diameter cold snare technique for the treatment of huge phytobezoars.

**Methods:**

This retrospective study enrolled 24 patients with huge phytobezoars treated with the cap-assisted 5-cm diameter cold snare technique in the Department of Gastroenterology, Nanchong Central Hospital, between December 25, 2022, and October 1, 2023. Patients' clinical characteristics and bezoar features were evaluated, the procedure was recorded, and patients were reviewed and followed with gastroscopy 1 day and 1 month after the procedure.

**Results:**

Twenty-four patients with huge phytobezoars were treated with the cap-assisted 5-cm diameter cold snare technique during the study period. The median phytobezoar size, break-up time, and extraction time were 5 × 3 cm (range 4–10, 3–5), 10.08 minutes (range 3.31–31.48), and 9.63 minutes (range 6.5–35.71), respectively. All patients achieved satisfactory treatment results, with no residual phytobezoars or gastrointestinal injuries on gastroscopy review 1 day after the procedure, and no postoperative adverse events were found on gastroscopy follow-up 1 month after the procedure.

**Conclusions:**

The results of this study indicated that the cap-assisted 5-cm diameter cold snare technique is safe, feasible, and effective for treating huge gastric bezoars, offering a new treatment method for this disease. Given the limitations inherent in the retrospective nature and relatively small sample size of this study, a prospective, multicenter, large-sample clinical trial is warranted to evaluate the efficacy and generalizability of this technique comprehensively.

## Introduction

Bezoars are formed by undigested substances retained in the stomach. They are classified into four types according to the ingested substances: phytobezoars, trichobezoars, pharmacobezoars, and lactobezoars [1]. Phytobezoars are the most common, consisting of hemicellulose, cellulose, tannins, and lignin [2]. Persimmon phytobezoars are the most common phytobezoars, which develop in people who frequently consume persimmons [3]. Clinical manifestations may differ depending on the size, hardness, and site of the bezoars, and a few cases maybe asymptomatic. Most of the patients experience abdominal pain, bloating, nausea, and vomiting, and those with huge bezoars may even develop life-threatening adverse events such as bleeding ulcers, intestinal obstructions, and intestinal perforations [1,4]. Current treatments for phytobezoars include chemical lysis, endoscopic removal, and surgical treatment [5]. However, persimmon phytobezoars are hard and can resist chemical dissolution [6,7], which often require endoscopic or surgical procedures. The current endoscopic treatments include simple snare, DualKnife, Ho:YAG laser, high-frequency currents, and argon plasma coagulation [8–12]. These treatments cannot totally remove large-diameter and hard bezoars; thus, surgical treatments are often required. However, patients do not easily accept surgical procedures because of trauma, high costs, and risk of postoperative adverse events [13]. Currently, there is a notable lack of effective endoscopic techniques for the management of large phytobezoars.

We innovatively apply the cap-assisted 5-cm diameter cold snare technique to quickly, effectively, and safely remove huge phytobezoars [14]. This study retrospectively analyzed 24 patients with huge phytobezoars and evaluated the feasibility, safety, and efficacy of the cap-assisted 5-cm diameter cold snare technique for removing huge phytobezoars.

## Patients and methods

### Study design

This single-center retrospective study was conducted in Nanchong Central Hospital, Sichuan Province. Informed consent was not required as it was a retrospective study and the data were analyzed anonymously. Authors hadn't access to information that could identify individual participants during or after data collection. Ethics committees specifically waived the need for consent. The study protocol was approved by its Medical Ethics Committee (No.2022093).

### Patients

This single-center retrospective study enrolled 24 patients with huge phytobezoars, who were admitted at the Department of Gastroenterology, Nanchong Central Hospital, between December 25, 2022, and October 1, 2023. Phytobezoars with a diameter greater than or equal to 3 cm are defined as huge phytobezoars. Those who were in poor physical condition (e.g., severe anemia, coagulation disorders, severe infections, severe cardiopulmonary dysfunction, and cannot tolerate general anesthesia) and could not tolerate gastroscopy were excluded. They were treated with the cap-assisted 5-cm diameter cold snare technique and underwent gastroscopy review 1 day and gastroscopy follow-up 1 month after the procedure.

### Technique description

An experienced endoscopist performed this technique. All patients completed preoperative investigations such as blood routine, urine routine, stool routine, liver function, kidney function, electrolytes, coagulation function, electrocardiogram, chest CT, cardiac ultrasound, and abdominal ultrasound to assess their general condition. All patients were fasted for 6–8 hours prior to operation and were treated under general anesthesia.

**Fig 1:**
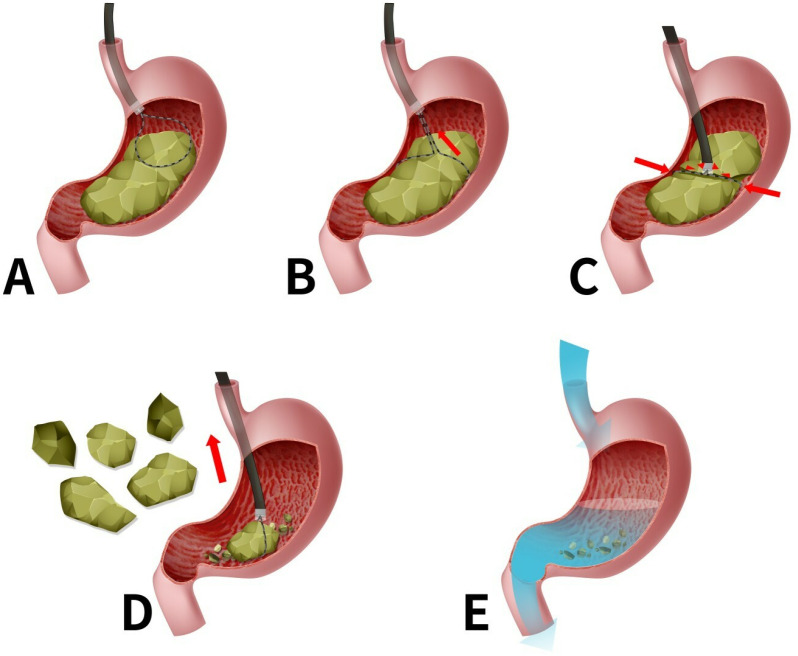
Mechanisms of the cap-assisted 5-cm diameter cold snare technique. **(A)** The 5-cm diameter snare is pushed forward to trap the huge bezoar completely. **(B)** Pulling the tightened snare backward brings the huge bezoar close to the white cap. **(C)** Cold-cutting the huge bezoar using the snare's pulling force and the cap's reaction force. **(D)** Larger bezoar pieces were removed using the 5-cm diameter snare. **(E)** Smaller bezoar pieces were washed out of the body by polyethylene glycol orally.

The cap-assisted 5-cm diameter cold snare technique included five steps, as shown in Fig 1.

**Step 1: Trapping the huge bezoar** (Fig 1A). The 5-cm diameter snare was inserted into the stomach through the endoscopic channel. The snare was expanded into a circle to obtain those with maximum diameters, and huge bezoars were trapped entirely with the snare. Compared with traditional snares, the 5-cm diameter snare can easily trap large-diameter bezoars.

**Step 2: Pulling the huge bezoar close to the white cap** (Fig 1B). Pulling the tightened snare backward brings the huge bezoar close to the white cap.

**Step 3: Cold-cutting of the huge bezoar** (Fig 1C). By further pulling the snare backward, using the snare's backward pulling force and the white cap's reaction force, the white cap successfully cuts the huge bezoar into small pieces (<2 cm) like a cold-cut cutter.

**Step 4: Extracting larger pieces** (Fig 1D). Larger bezoar pieces (1–2 cm) were removed using the snare without digestive tract injuries.

**Step 5: Washing out the leftover smaller pieces** (Fig 1E). The remaining smaller bezoar pieces (<1 cm) were flushed out of the body on the same day by polyethylene glycol orally (polyethylene glycol 137.5 g in 2000 mL of warm water).

### Video description

**Fig 2:**
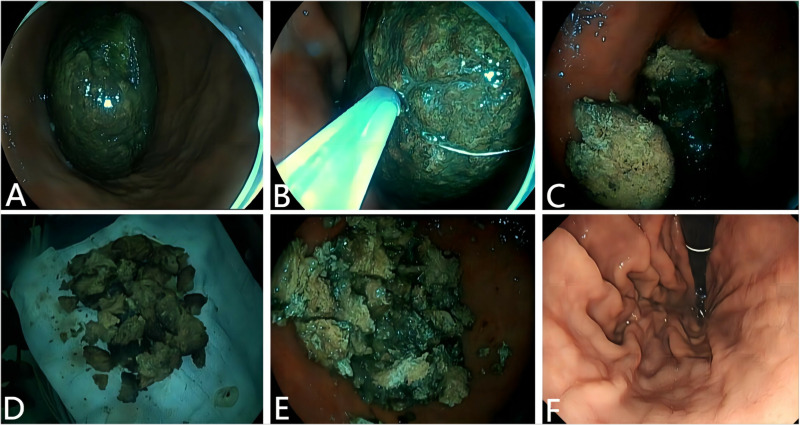
Cap-assisted 5-cm diameter cold snare technique in treating a huge bezoar in a 48-year-old female patient. **(A)** Gastroscopy showed a huge black bezoar (approximately 10 × 5 cm) in the stomach. **(B)** A 5-cm diameter snare was used to trap the huge bezoar. **(C)** The huge bezoar was successfully cut in half using the cap-assisted 5-cm diameter cold snare technique. **(D)** After repeated extractions, the snare removed the larger pieces. **(E)** The remaining smaller bezoar pieces were washed out of the body by polyethylene glycol orally. **(F)** Gastroscopy review 1 day after the procedure without residual bezoars or gastrointestinal injuries.

S1 Video: A 48-year-old woman was admitted to our department with a 1-month history of abdominal pain and a 2-month history of eating persimmons (Fig 2). Gastroscopy revealed a huge black phytobezoar measuring approximately 10 × 5 cm in the stomach (Fig 2A). The cap-assisted 5-cm diameter cold snare technique was used to treat this patient with a huge phytobezoar (S1 Video). A 5-cm diameter snare was used to trap the huge phytobezoar completely (Fig 2B), and the tightened snare was pulled back to the white cap to assist in cutting efficiently the huge phytobezoar into two pieces (Fig 2C). After repeated cap-assisted cold cuts, the huge phytobezoar was finally cut into pieces. Larger pieces were removed using the snare (Fig 2D), whereas smaller pieces were flushed out of the body with polyethylene glycol orally (Fig 2E). A gastroscopy review 1 day after the procedure showed that the huge phytobezoar was totally removed without causing digestive tract injuries (Fig 2F).

**Fig 3:**
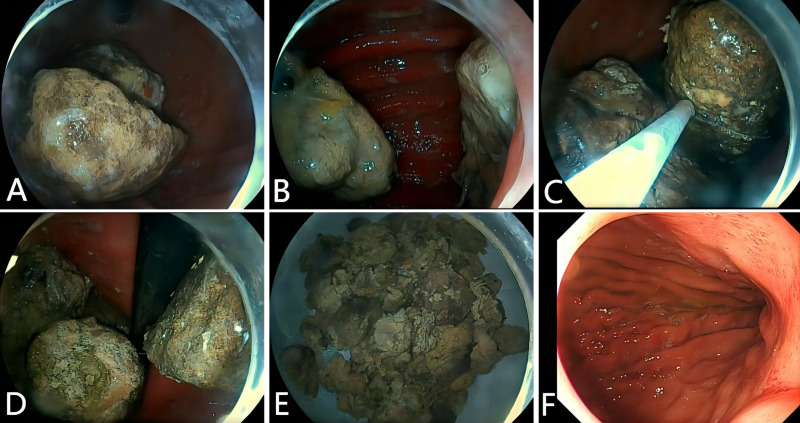
Cap-assisted 5-cm diameter cold snare technique in the treatment of four huge bezoars in a 71-year-old female patient. **(A** and **B)** Gastroscopy showed four huge brown bezoars (the largest was approximately 8 × 4 cm) in the stomach. **(C)** A 5-cm diameter snare was used to trap the huge bezoars. **(D)** The huge bezoar was successfully cut in half using the cap-assisted 5-cm diameter cold snare technique. **(E)** After repeated extractions, the snare removed the larger pieces. **(F)** Gastroscopy review 1 day after the procedure without residual bezoars or gastrointestinal injuries.

S2 Video: A 71-year-old woman was admitted to our department with a 1-month history of abdominal pain and a 1-month history of eating persimmons (Fig 3). Gastroscopy revealed four large, brown phytobezoars in the stomach, and the largest was approximately 8 × 4 cm (Fig 3A and B). The cap-assisted 5-cm diameter cold snare technique was used to treat this patient with four huge phytobezoars (S2 Video). A 5-cm diameter snare was used to trap the huge phytobezoars completely (Fig 3C), and the tightened snare was pulled back to the white cap to assist in cutting efficiently the huge phytobezoars into two pieces (Fig 3D). After repeated cap-assisted cold cuts, the huge phytobezoars were finally cut into pieces. Larger pieces were removed using the snare (Fig 3E), whereas smaller ones were flushed out of the body using polyethylene glycol orally. A gastroscopy review 1 day after the procedure showed that the huge phytobezoars were totally removed without causing digestive tract injuries (Fig 3F).

### Observe indices

The characteristics of the patients, namely, sex, age, symptoms, admission diagnosis, past medical history, surgical history, bezoar color, and bezoar size were collected. The relationship between the color and hardness of bezoars was compared. The break-up time and extraction time were recorded. The break-up time was defined as the time taken from the insertion of the 5-cm diameter snare to cold-cut huge bezoar into small pieces, and the extraction time was defined as the time taken to remove larger pieces from the body using the snare. Residual bezoar, gastrointestinal injury, and postoperative adverse events such as intestinal obstruction and gastrointestinal bleeding were also assessed.

### Statistical analysis

Statistical analyses were performed using SPSS version 28.0. Continuous variables are presented as median (range), while categorical variables are summarized as frequency counts and percentages.

## Results

### Characteristics of patients and color of bezoars

During the study period, 24 patients with huge bezoars received treatment using the cap-assisted 5-cm diameter cold snare technique. The characteristics of patients and color of bezoars are shown in Table 1 and S1 Table. The patients were 15 male and 9 female, aged 48–87 years, with a median age of 64 years. The majority of the patients were admitted for gastric ulcers (n = 17), and the remaining patients had dyspepsia (n = 4) or upper gastrointestinal bleeding (n = 3). All patients initially presented with abdominal pain (n = 24), some had nausea (n = 9) and vomiting (n = 4), and those with gastrointestinal bleeding vomited blood (n = 1) and had black stools (n = 2). All patients developed gastric ulcers of different degrees due to the compression of the gastric mucosa by gastric bezoars (n = 24). The majority of the patients had a history of eating persimmons months before admission (n = 10), most of these patients had black gastric bezoars (n = 11), and the rest had brown gastric bezoars (n = 13). Two patients had a history of gallbladder stone surgery, and two had a history of esophageal cancer radical surgery.

**Table 1:** Characteristics of patients and color of bezoars.

Patient No.	Sex	Age (y)	Symptoms	Admission diagnosis	Gastric ulcer/Forrest classification	Persimmon consumption/ duration time (m)	Medical history of affecting gastric motility	Bezoars color
1	M	57	Abdominal pain, nausea	Indigestion	Yes/III	No	No	Brown
2	F	79	Abdominal pain, tarry stool, nausea	Gastrointestinal bleeding	Yes/IIb	No	No	Black
3	F	54	Abdominal pain, vomiting	Gastric ulcer	Yes/Ib	Yes/4	Gallbladder stone surgery	Black
4	F	67	Abdominal pain	Gastric ulcer	Yes/IIc	Yes/3	No	Black
5	M	57	Abdominal pain, nausea	Gastric ulcer	Yes/III	Yes/1	No	Brown
6	M	79	Abdominal pain, nausea	Gastric ulcer	Yes/III	No	No	Brown
7	F	59	Abdominal pain, nausea	Indigestion	Yes/III	Yes/3	No	Black
8	M	52	Abdominal pain, nausea	Gastric ulcer	Yes/IIc	Yes/2	No	Brown
9	M	70	Abdominal pain, hematemesis	Gastrointestinal bleeding	Yes/Ib	No	Esophageal cancer radical surgery	Brown
10	M	78	Abdominal pain, tarry stool	Gastrointestinal bleeding	Yes/Ib	No	No	Brown
11	M	54	Abdominal pain, nausea, vomiting	Gastric ulcer	Yes/III	Yes/4	No	Black
12	M	57	Abdominal pain	Indigestion	Yes/III	No	No	Brown
13	F	71	Abdominal pain, nausea, vomiting	Gastric ulcer	Yes/III	Yes/1	Gallbladder stone surgery	Brown
14	F	48	Abdominal pain	Gastric ulcer	Yes/IIc	Yes/3	No	Black
15	M	64	Abdominal pain	Gastric ulcer	Yes/IIc	No	No	Brown
16	M	49	Abdominal pain	Indigestion	Yes/III	No	No	Black
17	F	52	Abdominal pain Bloating	Gastric bezoar Gastric ulcer	Yes/IIb	Yes/3	No	Black
18	M	76	Abdominal pain Bloating	Gastric bezoar Gastric ulcer	Yes//Ib	No	No	Black
19	F	64	Abdominal pain	Gastric bezoar Gastric ulcer	Yes/IIc	No	No	Brown
20	M	76	Abdominal pain Bloating	Gastric bezoar Gastric ulcer	Yes/Ib	No	No	Brown
21	M	87	Abdominal pain Bloating	Gastric bezoar Gastric ulcer	Yes/III	No	No	Brown
22	M	49	Abdominal pain	Gastric bezoar Gastric ulcer	Yes/IIc	No	No	Black
23	M	79	Abdominal pain Bloating	Gastric bezoar Gastric ulcer	Yes/III	Yes/3	No	Black
24	F	67	Abdominal pain Bloating vomiting	Gastric bezoar Gastric ulcer	Yes/IIc	No	Esophageal cancer radical surgery	Brown

Note: F = Female; M = Male; y = year; m = month

### Bezoar size, break-up time, extraction time, review and follow-up results

All 24 patients were successfully treated using the cap-assisted 5-cm diameter cold snare technique. The bezoar size, break-up time, extraction time, review and follow-up results of the patients are shown in Table 2 and S1 Table. The median bezoar size was 5 × 3 cm (range 4–10, 3–5), and the largest one was 10 × 5 cm. Four huge bezoars (8 × 4 cm, 6 × 4 cm, 5 × 4 cm, and 3 × 3 cm) were found in the stomach of one patient, and the most of the patients had only one huge bezoar in their stomach (n = 21). The median break-up and extraction times were 10.08 minutes (range 3.31–31.48) and 9.63 minutes (range 6.5–35.71), respectively. The patient with four huge bezoars in the stomach had longer break-up (31.48 min) and extraction (35.71 min) times. All other patients had break-up and extraction times of < 16 min. The gastroscopy review 1 day after the procedure did not reveal any residual bezoars or digestive tract injuries, and no postoperative adverse events were observed at the 1-month gastroscopy follow-up.

**Table 2:** Bezoar size, break-up time, extraction time, review and follow-up results.

Patient No.	Sex	Age (y)	Bezoars size (cm)	Break-up time (min)	Extraction time (min)	One-day review/ digestive tract injury	One-day review/ bezoar residue	One-month follow-up/ postoperative adverse events
1	M	57	8 × 3	12.36	13.28	NO	NO	NO
2	F	79	5 × 4	10.46	10.33	NO	NO	NO
3	F	54	10 × 4	13.25	14.68	NO	NO	NO
4	F	67	4 × 3	5.43	8.13	NO	NO	NO
5	M	57	4 × 3	9.70	8.18	NO	NO	NO
6	M	79	6 × 4	13.23	10.53	NO	NO	NO
7	F	59	10 × 5	14.66	14.13	NO	NO	NO
8	M	52	5 × 3	12.11	9.91	NO	NO	NO
9	M	70	4 × 3	3.31	7.85	NO	NO	NO
10	M	78	4 × 3	4.85	8.31	NO	NO	NO
11	M	54	5 × 3	7.71	8.80	NO	NO	NO
12	M	57	5 × 3	7.85	8.66	NO	NO	NO
13	F	71	8 × 4, 6 × 4, 5 × 4, 3 × 3	31.48	35.71	NO	NO	NO
14	F	48	10 × 5	14.86	14.35	NO	NO	NO
15	M	64	4 × 4	5.45	8.51	NO	NO	NO
16	M	49	5 × 3	6.81	9.35	NO	NO	NO
17	F	52	8 × 5	12.52	12.46	NO	NO	NO
18	M	76	5 × 3	9.35	8.68	NO	NO	NO
19	F	64	7 × 4 6 × 4	15.18	15.72	NO	NO	NO
20	M	76	5 × 3 4 × 3	13.58	12.44	NO	NO	NO
21	M	87	5 × 3	7.12	8.31	NO	NO	NO
22	M	49	8 × 4	11.84	12.55	NO	NO	NO
23	M	79	4 × 3	5.85	7.25	NO	NO	NO
24	F	67	4 × 3	5.33	6.50	NO	NO	NO

Note: F = Female; M = Male; y = year

## Discussion

**Fig 4:**
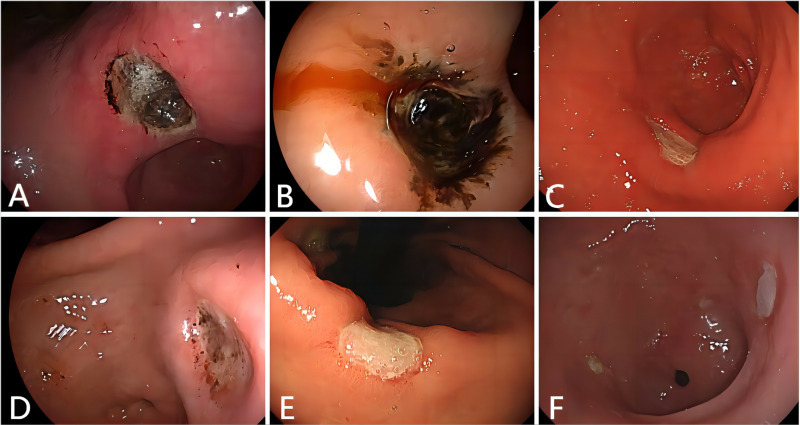
Gastric ulcers are caused by chronic compression of the gastric mucosa by gastric bezoars. **(A)** A deeply concave ulcer with a black base in the gastric angle (Forrest IIb). **(B)** A superficial ulcer with an attached blood clot and slight active bleeding in the anterior wall of the gastric sinus (Forrest Ib). **(C)** A superficial concave ulcer with a clean base covered and a white coating in the gastric sinus (Forrest III). **(D)** A superficial concave ulcer covered with a white coating and few blood clots on the body of the stomach (Forrest IIb). **(E)** A superficial concave ulcer with a clean base covered and white coating in the gastric angle (Forrest III). **(F)** A superficial concave ulcer with a clean base covered and white coating in the gastric sinus (Forrest III).

Older patients are more likely to suffer from gastric bezoars because of delayed gastric emptying caused by reduced gastrointestinal motility [8]. In this study, the patient’s age ranged from 48 to 87 years, with a median age of 64 years, including thirteen older patients (Table 1 and S1 Table). In addition, some patients with a history of previous gastrointestinal surgery were more likely to develop gastric bezoars. These gastrointestinal surgeries, such as esophageal cancer radical surgery, partial gastrectomy, vagotomy, and pyloroplasty, may result in anastomotic stenosis, decreased gastric acid, gastric retention, and pyloric stenosis, causing the retention of some indigestible materials in the stomach forming gastric bezoars [1]. In this study, two patient with a history of surgery for esophageal cancer had decreased gastrointestinal motility, which resulted in the formation of a gastric bezoar. Persimmon phytobezoars are the most common phytobezoars [15]. Our study also found ten patients with long-term consumption of persimmons, which caused gastric bezoars without gastrointestinal dysmotility diseases. Patients’ clinical symptoms depend on the size and location of gastric bezoars, with abdominal pain, nausea, and vomiting as the main symptoms [4]. Bezoar’s long-term compression of the gastric mucosa resulting in bleeding gastric ulcer may also cause hematemesis, black stools, and other upper gastrointestinal bleeding symptoms [16]. Thirteen patients in this study were admitted for abdominal pain and three for upper gastrointestinal bleeding. When gastric bezoars enter the small intestines, the larger ones can cause complete or partial mechanical intestinal obstruction, intestinal perforation or even peritonitis, and other acute abdominal conditions [17,18]. The compression of the gastric mucosa by gastric bezoars often causes gastric ulcers [19]. In this study, all 24 patients had different degrees of gastric ulcer (Fig 4), including five cases of Forrest Ib, ten cases of III, seven cases of IIc, and two case of IIb.

Persimmon phytobezoars are usually black or darkish brown, and the surface is generally black and extremely hard, whereas the inside is typically yellowish brown and relatively soft [20]. Several clinical reports have highlighted the suboptimal performance of endoscopic techniques in managing persimmon phytobezoars [15,21]. However, our study had different findings, i.e., the color and hardness of phytobezoars may be related to the duration of persimmon consumption. Among the ten patients who had eaten persimmons, the phytobezoars were brown and relatively soft in three patients who had eaten persimmons for < 2 months, whereas the phytobezoars were black and hard on the outside and inside in seven patients who had eaten persimmons for > 3 months. Our clinical research demonstrated that persimmon phytobezoars in patients with short-term persimmon ingestion typically manifest as brown with a relatively friable surface, making them amenable to mechanical fragmentation. In contrast, those in patients with prolonged persimmon ingestion often present as black, characterized by a hardened surface, exhibiting significant resistance to mechanical fragmentation.

Current endoscopic treatments for huge bezoars include simple snare, DualKnife, Ho:YAG laser, high-frequency currents, and argon plasma coagulation [8–12]. With simple snares [9], large-diameter gastric bezoars are difficult to trap because conventional small-diameter snares are used, which can only cut smaller and softer bezoars. Although DualKnife [10] can cut gastric bezoars; however, it is inefficient, takes more time, and may damage the mirror body. Ho:YAG laser [11] has a low cutting efficiency and takes time. Argon plasma coagulation [12] can only loosen the inside of gastric bezoars and cannot break it up alone, which must be used in conjunction with a snare or other tools to complete the fragmentation. Recently, some researchers focusing on the endoscopic treatment of huge gastric bezoars reported the use of a guidewire to remove bezoars [22]. However, the use of special instruments is not common, which is not beneficial to promote its application. Non-invasive treatment usually uses chemical dissolution. Although Cola has a specific chemical dissolution effect and can dissolve some small and loose gastric bezoars, phytobezoars are difficult to dissolve because of their hard consistency [6,7,23], and bezoars may fall into the intestine during the dissolution process, increasing the risk of intestinal obstruction. Thus, huge bezoars usually require surgical treatment [24]. However, surgical procedures can be more traumatic for patients and may have adverse events such as postoperative infection, postoperative bleeding, and incisional hernia [13], which increase the risks for patients; thus, they barely accept them. Therefore, the cap-assisted 5-cm diameter cold snare technique proposed in this study has significant clinical implications for treating huge bezoars.

We used a 5-cm diameter snare to trap huge gastric bezoars easily, and a white cap was attached to the end of the gastroscope as a support to assist in cold-cutting bezoars [14]. We used the force of the pulling snare and the reaction force of the white cap, which can cut huge bezoars safely and efficiently, like a cold-cutter. In this retrospective study, all 24 patients with huge bezoars were treated successfully, with median break-up and extraction times were 10.08 minutes (range 3.31–31.48) and 9.63 minutes (range 6.5–35.71). Huge gastric bezoars were cold-cut using a 5-cm diameter snare, larger pieces were removed with the snare, and the remaining smaller pieces were washed out of the body using polyethylene glycol orally. The natural excretion of bezoars is associated with a risk of re-gathering residual pieces due to the effect of gastric acid [25]. We used polyethylene glycol to wash out the remaining smaller pieces, effectively avoiding this risk. Thus, no residual bezoars or gastrointestinal injuries were found on gastroscopy review 1 day after the procedure, and no postoperative adverse events occurred at the 1-month gastroscopy follow-up.

The technique demands an experienced endoscopist, and this could potentially pose a challenge to its widespread adoption in centers with limited expertise. However, we have several strategies in mind to address this issue. First, we plan to develop a comprehensive training program specifically tailored for this technique. This program will cover theoretical knowledge, hands - on training in a simulated environment, and supervised clinical practice. Second, we intend to establish a mentorship network where experienced endoscopists can provide guidance and support to their counterparts in less - experienced centers. This can be achieved through regular video - conferencing consultations, on - site visits, and case - based discussions. Finally, We are confident that these efforts will gradually reduce the expertise - related barriers and facilitate the wider application of the technique across different centers.

### Limitations

This study has several limitations that warrant consideration. First, the single-center design may restrict the generalizability of our findings to other institutions or diverse patient populations. Second, the retrospective nature of the study introduces potential selection bias and inherent systematic errors that are challenging to fully mitigate. Third, the relatively small sample size (n = 24) may limit the statistical power to comprehensively characterize population-wide features. To address these constraints, we plan to conduct a prospective, multicenter, large-sample randomized controlled study to validate the broader applicability of the technique. We also plan to incorporate a cost-effectiveness analysis to provide a more comprehensive assessment of the economic benefits of the technology.

## Conclusion

In conclusion, this retrospective study proved the feasibility, safety, and efficiency of the cap-assisted 5-cm diameter cold snare technique for treating huge gastric bezoars, offering a new treatment method for this disease. Given the limitations inherent in the retrospective nature and relatively small sample size of this study, a prospective, multicenter, large-sample clinical trial is warranted to evaluate the efficacy and generalizability of this technique comprehensively.

## Supporting information

S1 Video The cap-assisted 5-cm diameter cold snare technique was used to treat the patient with a huge black phytobezoar. (https://drive.google.com/file/d/1VhVf_yl8pVK8D9GUP66eL1xWH7HUjdm8/view?usp=drive_link)
(MOV)

S2 Video The cap-assisted 5-cm diameter cold snare technique was used to treat the patient with four huge brown phytobezoars. (https://drive.google.com/file/d/13HQZWT2Wmxe9ddvw82zPjUV6PjH4Oxer/view?usp=drive_link)
(MOV)

S1 Table Characteristics, operative details, results, and follow-up outcomes of patients. (DOCX)
