# Supplementary material for: Cap-assisted 5-cm diameter cold snare treatment for phytobezoars: A retrospective study
Source: PLoS One. 2025 May 7;20(5):e0323226. doi: 10.1371/journal.pone.0323226 (PMC12058175; doi:10.1371/journal.pone.0323226)
Supplement: S1 Table — (DOCX) [file pone.0323226.s003.docx]

| **S1 Table. Characteristics, operative details, results, and follow-up outcomes of patients** | |
| --- | --- |
| **Age, median (range), years** | 64 (48-87) |
| **Sex, n (%)** |  |
| Male | 15 (62.5) |
| Female | 9 (37.5) |
| **Symptoms, n (%)** |  |
| Abdominal pain | 24 (100) |
| nausea | 9 (37.5) |
| vomiting | 4 (16.7) |
| hematemesis | 1 (4.2) |
| tarry stool | 2 (8.3) |
| **Admission diagnosis, n (%)** |  |
| Gastric ulcer | 17 (70.8) |
| Indigestion | 4 (16.7) |
| Gastrointestinal bleeding | 3 (12.5) |
| **Gastric ulcer Forrest classification, n (%)** |  |
| I b | 5 (20.8) |
| II b | 2 (8.3) |
| II c | 7 (29.2) |
| III | 10 (41.7) |
| **Persimmon consumption, n (%)** |  |
| Duration: 1 month | 2 (8.3) |
| Duration: 2 month | 1 (4.2) |
| Duration: 3 month | 5 (20.8) |
| Duration: 4 month | 2 (8.3) |
| **Medical history of affecting gastric motility, n (%)** |  |
| Gallbladder stone surgery | 2 (8.3) |
| Esophageal cancer radical surgery | 2 (8.3) |
| **Bezoars color, n (%)** |  |
| Black | 11 (45.8) |
| Brown | 13 (54.2) |
| **Bezoars size, median (range), cm** | 5 (4-10) × 3 (3-5) |
| **Break-up time, median (range), minutes** | 10.08 (3.31-31.48) |
| **Extraction time, median (range), minutes** | 9.63 (6.5-35.71) |
| **One-day review , n (%)** |  |
| Digestive tract injury | 0 (0) |
| Bezoar residue | 0 (0) |
| **One-month follow-up , n (%)** |  |
| Postoperative adverse events | 0 (0) |
